# Heterogeneous Signaling at GABA and Glycine Co-releasing Terminals

**DOI:** 10.3389/fnsyn.2018.00040

**Published:** 2018-11-06

**Authors:** Karin R. Aubrey, Stéphane Supplisson

**Affiliations:** ^1^Institut de Biologie de l’Ecole Normale Supérieure (IBENS), Ecole Normale Supérieure, CNRS, INSERM, PSL Université Paris Paris, France; ^2^Neurobiology of Pain Laboratory, Kolling Institute, Royal North Shore Hospital St. Leonards, NSW, Australia; ^3^Pain Management Research Institute, Faculty of Medicine and Health, University of Sydney—Northern Clinical School St. Leonards, NSW, Australia

**Keywords:** GABA, glycine, inhibitory neurotransmission, VIAAT, VGAT, GlyT2-eGFP mouse, cotransmission, quantal release

## Abstract

The corelease of several neurotransmitters from a single synaptic vesicle has been observed at many central synapses. Nevertheless, the signaling synergy offered by cotransmission and the mechanisms that maintain the optimal release and detection of neurotransmitters at mixed synapses remain poorly understood, thus limiting our ability to interpret changes in synaptic signaling and identify molecules important for plasticity. In the brainstem and spinal cord, GABA and glycine cotransmission is facilitated by a shared vesicular transporter VIAAT (also named VGAT), and occurs at many immature inhibitory synapses. As sensory and motor networks mature, GABA/glycine cotransmission is generally replaced by either pure glycinergic or GABAergic transmission, and the functional role for the continued corelease of GABA and glycine is unclear. Whether or not, and how, the GABA/glycine content is balanced in VIAAT-expressing vesicles from the same terminal, and how loading variability effects the strength of inhibitory transmission is not known. Here, we use a combination of loose-patch (LP) and whole-cell (WC) electrophysiology in cultured spinal neurons of GlyT2:eGFP mice to sample miniature inhibitory post synaptic currents (mIPSCs) that originate from individual GABA/glycine co-releasing synapses and develop a modeling approach to illustrate the gradual change in mIPSC phenotypes as glycine replaces GABA in vesicles. As a consistent GABA/glycine balance is predicted if VIAAT has access to both amino-acids, we test whether vesicle exocytosis from a single terminal evokes a homogeneous population of mixed mIPSCs. We recorded mIPSCs from 18 individual synapses and detected glycine-only mIPSCs in 4/18 synapses sampled. The rest (14/18) were co-releasing synapses that had a significant proportion of mixed GABA/glycine mIPSCs with a characteristic biphasic decay. The majority (9/14) of co-releasing synapses did not have a homogenous phenotype, but instead signaled with a combination of mixed and pure mIPSCs, suggesting that there is variability in the loading and/or storage of GABA and glycine at the level of individual vesicles. Our modeling predicts that when glycine replaces GABA in synaptic vesicles, the redistribution between the peak amplitude and charge transfer of mIPSCs acts to maintain the strength of inhibition while increasing the temporal precision of signaling.

## Introduction

A well-established and long accepted postulate known as “Dale’s principle” proposes that neurons have chemical unity for signaling (Strata and Harvey, 1999; Tritsch et al., 2016) —meaning that they communicate by releasing the same neurotransmitter at all their synapses. When the quantal nature of synaptic transmission was later discovered, application of Dale’s principle implied that each presynaptic terminal shares the particular set of enzymes and transporters that are required for the synthesis, recapture and accumulation of a specific neurotransmitter(s) into the synaptic vesicles, which are recycled and refilled locally before being reused (Hnasko and Edwards, 2012). Now it is clear that many central synapses corelease multiple neurotransmitters, expanding the repertoire of chemical signaling (Vaaga et al., 2014; Granger et al., 2017) and the extent of presynaptic coordination require to uphold Dale’s principal.

In the hindbrain, GABA and glycine are coreleased from single vesicles (Jonas et al., 1998; Lu et al., 2008). Although GABA and glycine have separate presynaptic supply mechanisms, postsynaptic receptors, and modulators, they share the same vesicular transporter, VIAAT (Wojcik et al., 2006; Aubrey et al., 2007) and when GABA/glycine are coreleased from the same single vesicle, mixed miniature inhibitory postsynaptic currents (mIPSCs) have a characteristic biphasic time course (Jonas et al., 1998; Keller et al., 2001; Russier et al., 2002). The regulation of GABA/glycine corelease is known to play a significant role during the maturation of spinal inhibitory circuits (Chéry and de Koninck, 1999; Keller et al., 2001; Russier et al., 2002; Coull et al., 2003; Rahman et al., 2013; Medelin et al., 2016), as well as in other hind-brain regions including the auditory nucleus (Kotak et al., 1998; Lu et al., 2008; Fischl and Burger, 2014; Nerlich et al., 2014) and cerebellum (Dugué et al., 2005; Rousseau et al., 2012). Early biochemical examinations of GABA and glycine uptake into synaptosomes (an isolated synaptic terminal preparation) indicated that both amino acids compete for the same vesicular transporter (Fykse and Fonnum, 1988; Burger et al., 1991; Christensen and Fonnum, 1991). This idea was endorsed when VIAAT was found in both GABAergic and glycinergic terminals (McIntire et al., 1997; Sagné et al., 1997) and taken together, these data suggested that the vesicular content is determined by the relative occupancy of GABA/glycine at VIAAT (Gasnier, 2000) and therefore, will be primarily determined by the relative presynaptic cytosolic concentrations of the two inhibitory neurotransmitters.

In active terminals, GABA is synthesized from glutamate by two isoforms of the glutamate decarboxylase enzyme (GAD65/67; Martin and Rimvall, 1993; Kakizaki et al., 2015), whereas the concentrative power of GlyT2, a 3 Na^+^-coupled glycine transporter (Roux and Supplisson, 2000), is required to increase basal glycine concentration to the appropriate levels for VIAAT uptake (Gomeza et al., 2003; Rousseau et al., 2008; Apostolides and Trussell, 2013). Experimental evidence confirms that the cytosolic concentration of glycine and GABA does indeed influence synaptic vesicle content (Rousseau et al., 2008; Apostolides and Trussell, 2013; Ishibashi et al., 2013). Furthermore, two *in* *vitro* studies have succeeded in sampling the inhibitory transmitter content of VIAAT-expressing vesicles at single terminals and found evidence that the mIPSCs phenotypes were not homogenous. Instead, GABA-, glycine- and mixed mIPSCs were all detected at some individual synapses, suggesting that the signaling phenotype of a single terminal may considerably vary from vesicle to vesicle (Katsurabayashi et al., 2004; Aubrey et al., 2007; Supplementary Figure S1).

Here, we record mIPSCs in networks of culture spinal cord neurons and extract the subpopulation of mIPSCs that originate from an individual terminal by simultaneously recording mIPSCs with a loose-cell patch-clamp electrode placed over a single pre-synaptic varicosity. Then, we examine whether vesicles originating from the same co-releasing terminal evoke homogenous postsynaptic currents, as expected if all of a presynaptic terminal’s vesicles contain a similar concentration of GABA and glycine. In addition, we develop a simulation model of cotransmission to examine how mIPSC peak amplitude and charge transfer change as function of glycine vs. GABA release. Using activation kinetic schemes of GlyR and GABA_A_R, our simulations show that alterations in vesicular content would not compromised the strength, but rather would shape the time-course of postsynaptic inhibition.

## Materials and Methods

### Embryonic Mouse Spinal Cord Neurons

Primary cultures of spinal cord neurons were prepared as described in Hanus et al. (2004) from embryonic day 13 or 14 (E13–14) C57BL/6J wild-type or heterozygous GlyT2-EGFP mouse pups (Zeilhofer et al., 2005). Embryos were obtained by cesarean section from pregnant mice anesthetized by intraperitoneal injection of ketamine-xylazine (100 and 10 mg/kg) and killed by cervical dislocation. Spinal cords were dissected under sterile conditions into PBS with 33 mM glucose at pH = 7.4 and then incubated in trypsin/EDTA solution (0.05% v/v, Sigma, St. Louis, MO, USA) for 10 min at 37°C. Cells were dissociated mechanically in a modified L15 Leibowitz’s medium (Life Technologies, Cergy Pontoise, France) and plated at a density of 1.0 × 10^5^ cells/cm^2^ on sterilized glass coverslips coated with 60 μg/ml poly D-L ornithine and with medium containing 5% inactivated fetal calf serum (Sigma, St. Louis, MO, USA). To insure easy visualization of a few eGFP-GlyT2 positive axons and their boutons, each coverslip was composed of 5%–10% neurons from GlyT2-eGFP mice; the remaining 90%–95% neurons were from unlabeled WT littermates. Cells were maintained at 37°C in 5% CO_2_ in serum-free Neurobasal™ medium containing supplement B27 (Invitrogen, Carlsbad, CA, USA; Brewer et al., 1993) for up to 3 weeks. Medium was changed every 4–5 days.

### Electrophysiology

Whole-cell (WC) patch clamp recordings of spinal cord neurons (14–22 DIV) were performed at 30°C. Voltage-clamp was imposed by a Multiclamp 700B amplifier controlled by pCLAMP 9 or 10 acquisition software (Molecular Devices). Currents were filtered at 4 kHz and sampled at 20 kHz using a Digidata 1440A (Molecular Devices). Neurons were continuously bathed with an external solution containing (mM): NaCl 140, KCl 2.4, CaCl_2_ 2, MgCl_2_ 2, Glucose 10, HEPES 10, pH = 7.4. WC patch clamped mIPSCs were recorded in the presence of 0.2 μM of the sodium channel blocker tetrodotoxin (TTX) and 2 μM of the benzodiazepine flunitrazepam. AMPA and NMDA receptors were blocked with 2 μM NBQX (6-nitro-7-sulfamoylbenzo[f]quinoxaline-2, 3-dione) and 5 μM MK-801, respectively. When indicated GABA_A_ receptors (GABA_A_Rs) were blocked with 5 μM gabazine (SR 95531), and glycine receptors (GlyRs) were blocked with 1 μM strychnine. The majority of whole cell mIPSCs have fast rise times, and a few cells were found to have a correlation between their glycinergic mIPCS rise and decay time constants, suggesting that dendritic filtering of mIPSCs from more distant synapses influences the kinetic of some, but not all, events included in the WC mIPSC data (Supplementary Figure S2C; Gardner et al., 1999). There was no evidence of dendritic filtering at any of the single synapse mIPSCs, as expected for events that originated from synaptic terminals that are on, or very close to, the neuronal soma (Supplementary Figure S2C).

Patch-pipettes were pulled from borosilicate glass capillaries (Hilgenberg, Maisfeld, Germany) and pipettes for WC recording had typical resistances of 4–6 MΩ. The mIPSCs were recorded at a holding potential (V_H_) of −70 mV (taking into account the junction potential) using pipettes filled with a standard internal solution containing (in mM): CsCl 140, CaCl_2_ 1, EGTA 10, BAPTA 1, MgCl_2_ 1, Mg-ATP 4, QX314-Cl 5, Hepes 10, adjusted to pH 7.4 with CsOH. Loose-patch (LP) recording pipettes had resistances of 1–2 MΩ and LP mIPSCs were recorded at 0 mV. One out of every 10 attempts at obtaining a LP recording were successful. LP pipettes were filled with the high calcium external solution (4 mM Ca^2+^; 0 mM Mg^2+^), 0.2 μM TTX, 2 μM flunitrazepam, 2 μM NBQX, 5 μM MK-801. Baclofen, a GABA_B_R agonist, was added to the extracellular solution to reduce the frequency of mIPSC when required (O’Brien et al., 2004). Unless indicated, drugs were purchased from Sigma (St. Louis, MO, USA) or Tocris Bioscience (Bristol, UK).

### Data Analysis

Miniature synaptic currents were detected semi-automatically and analyzed using SpAcAn, a custom-made integral detection Igor package developed by Guillaume Dugué and Charly Rousseau and available at www.spacan.net. or in AxoGraph. LP and WC mIPSCs whose peak currents occurred simultaneously (<0.6 ms) and were verified to have a smooth rise and decay phase, as well as a strongly correlated peak amplitudes, were included in the analysis. Electrophysiological results are reported as mean ± SEM. All statistical tests were nonparametric and performed using Prism software unless indicated. The Mann–Whitney and Wilcoxon Matched-Pairs test was used to assess differences between two independent and two related samples respectively. For all tests, the number of asterisks (*) in the figures corresponds to level of significance: ***p < 0.001, **p < 0.01 and *p < 0.05.

Populations of glycine and GABA mIPSCs recorded in the presence of SR 95331 or strychinine, respectively, were classified as glycinergic, GABAergic or mixed on the basis of their peak amplitude to charge transfer relationship and then fitted with a linear regression and 95% prediction intervals. We also attempted to classify mIPSC events by fitting their currents with biexponential and monoexponential fits (Jonas et al., 1993; Rahman et al., 2013) but found that the peak amplitude to charge relationship robust and fast (Supplementary Figure S2). We set a conservative limit of ≥30% to considering groups of single synapse mIPSCs as a separate population. This value was based on our pharmacological characterization of glycine and GABA mIPSCs.

### Numerical Simulation

The set of linear differential equations that defined the GlyR and GABA_A_R kinetic models was numerically solved in Mathematica 11 using the Q-matrix approach (Colqhoun and Hawkes, 1995). The current amplitude corresponds to the summation of 120 GlyRs and 60 GABA_A_Rs, with elementary currents of 1.05 and 3 pA for the GABA_A_R mono- and di-liganded receptors, respectively, and 5 pA for GlyR. The rate constants are indicated in Figure 3A, that correspond to the values determined by Burzomato et al. (2004) for GlyR and Labrakakis et al. (2014) for GABA_A_R except for the dissociation rate constants that were slightly increased to match the experimental time course of the mIPSC and peak-charge amplitude relationship.

**Figure 1:**
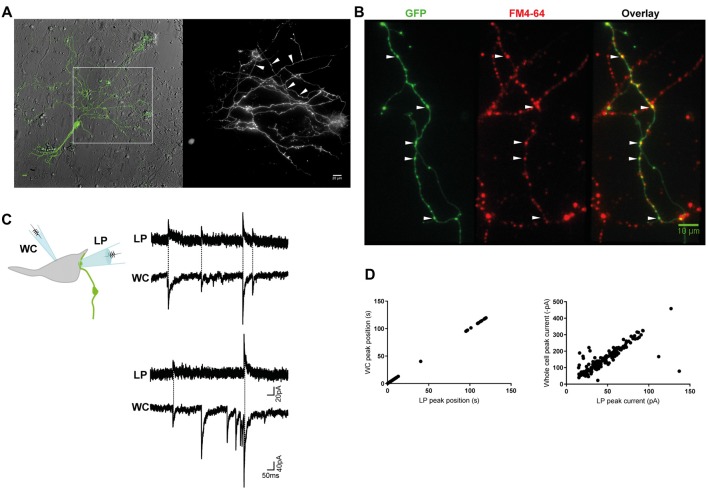
Miniature inhibitory postsynaptic currents (mIPSCs) were recorded from individual inhibitory terminals. **(A)** GlyT2-eGFP^+^ neurons and neurites were visible in our spinal cord cultures. eGFP^+^ neurites with a parallel structure and beaded varicosities (*) were labeled with FM 4–64 dye **(B)**, confirming they are glycine-containing presynaptic terminals. Scale bars are 10 μm. **(C)** Cartoon of the paired recording configuration that allowed identification of mIPSCs that originated from an individual inhibitory terminal (top). Postsynaptic mIPSCs were simultaneously recorded with the whole-cell patch clamp pipette (WC) and the loose-patch clamped pipette (LP). The traces (middle and bottom) are a continuous 2 s recording from one WC-LP pair. **(D)** The LP and whole cell mIPSCs from the same recording as displayed in **(C)**, were strongly correlated in time and amplitude, as expected from an extracellular and intracellular measurement of the same signal.

**Figure 2:**
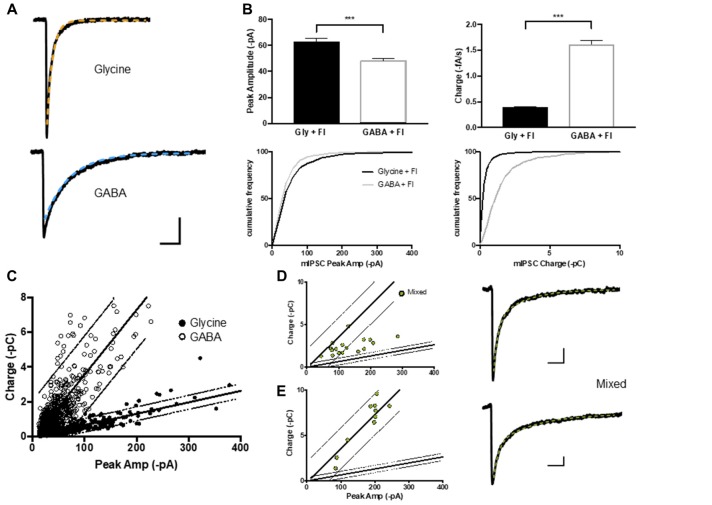
GABA, glycine and mIPSCs were defined on the basis of their peak current and charge transfer. **(A)** Average current traces of glycinergic and GABAergic mIPCs were recorded in whole-cell configuration in the presence strychnine or SR95331 and decays fitted with a single exponential (orange and blue) respectively. **(B,C)** The peak current (I) to charge ratio of GABA and glycine mIPSC (*n* = 600 events from 15 neurons each) were used to define 95% prediction intervals (gray lines) for these two mIPSC phenotypes. **(D,E)** Two example populations of mixed mIPSCs recorded in the absence of strychnine or SR 95331 and selected on the basis of their biphasic decay current (double exponential fit (green)). Mixed mIPSCs mapped between the two 95% prediction intervals or were misidentified as GABAergic, depending on the size of their GABA component.

**Figure 3:**
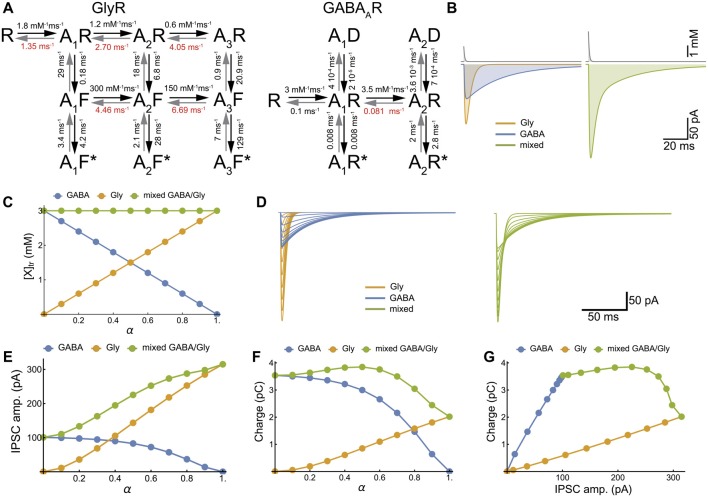
Model of cotransmission shows that swapping glycine for GABA in the neurotransmitter transient maintains the strength of synaptic inhibition. **(A)** Kinetic schemes for the activation of GlyR (Burzomato et al., 2004) and GABA_A_R (Jones and Westbrook, 1995; Labrakakis et al., 2014) that were used for the numerical simulations. We adopted the set of rate constants determined by Burzomato et al. (2004) and Labrakakis et al. (2014); with the exception of the dissociation rate constants, indicated in red, that were adjusted for matching the time course of our recorded mIPSCs (τ = 6.4 ms and 34.3 ms for glycine and GABA IPSC respectively). **(B)** Numerical simulations of the mIPSCs evoked by concentration transient (1.5 mM) of glycine or GABA applied alone (left) or together (right). The concentration scale is the total concentration of neurotransmitter. **(C)** Inverse relationships of GABA and glycine concentrations for increasing values of α, which is the fraction of glycine released during each transient, assuming constant vesicular release (3 mM). **(D–G)** Numerical simulations of glycinergic, GABAergic and mixed mIPSCs when α increased from 0 to 1 by increments of 0.1, showing the current traces **(D)**, the IPSC peak amplitudes **(E)**, the IPSC charge transfer **(F)** and the IPSC charge-amplitude relationships **(G)**.

We used the simplified assumption of a monoexponential time course for the transient of GABA and glycine (AAN: amino-acid neurotransmitter) with a piecewise function:

$${\text{[AAN]}}_{t}\left\{ \begin{array}{l} 0\ldots \ldots \ldots \ldots \ldots \ldots \ldots \ldots \ldots \ldots \ldots \ldots ..{\text{if t < t}}_{0}\\ {\text{[AAN]}}_{peak}{\text{e}}^{-(t-t_{0})/\tau }\ldots \text{if t }\geq {\text{ t}}_{0} \end{array}\right.$$

The time constant of the transient in Figure 3 was τ = 0.7 ms and the [AAN]_peak_ = [Gly]_peak_ + [GABA]_peak_ = 3 mM (Beato, 2008). The time offset (t_0_) was 2 ms and the time of integration was 200 ms.

The simulated mixed mIPSC corresponds to the algebraic sum of the glycine and GABA mIPSCs. We used the FindMinimum and NIntegrate functions of Mathematica 11 (Wolfram Research) to determine the peak amplitude and charge transfer of simulated mIPSCs.

The parameter α set the relative glycine concentration in the transient:

$$\alpha =\frac{{\left[Gly\right]}_{tr}}{{\left[Gly\right]}_{tr}+{\left[GABA\right]}_{tr}}$$

**Figure 4:**
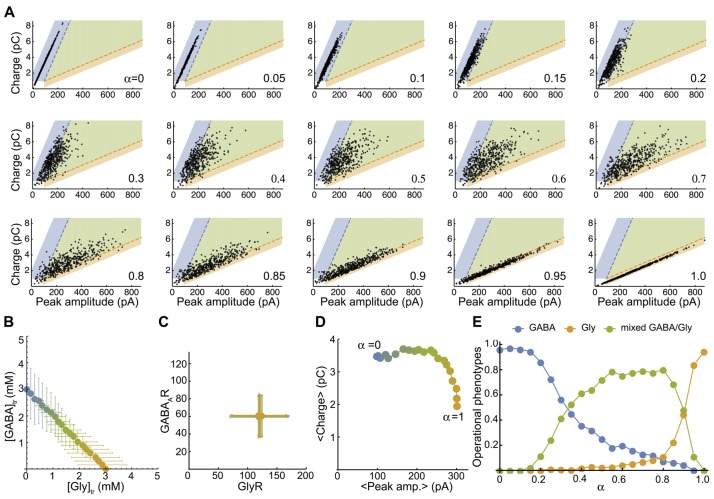
The simulated mixed mIPSC phenotypes shifts as function of the proportion of glycine and GABA coreleased. **(A)** Simulated-mIPSC charge-amplitude distribution for increasing α values. Each panel shows the charge-amplitude distribution of 500 randomly simulated mIPSCs for the α value indicated. We defined three areas as GABAergic (blue), glycinergic (orange) and mixed (green) to operationally sort individual mIPSC phenotypes with similar threshold criteria that were used experimentally. **(B)** Parametric plot of the average GABA-glycine peak concentration in the transient as function of α. Each value was randomly generated from normal distributions as function of α with CV_AAN_ = 0.4 ([gly]: 3 α ± 1.2 α mM and [GABA] = 3 (1−α) ± 1.2 (1-α) mM). **(C)** Plot of the average number of GABA_A_R-GlyR randomly generated from normal distributions with CV_R_ = 0.4 (120 ± 48 GlyRs and 60 ± 24 GABA_A_Rs). **(D)** Plot of the average charge-peak amplitude. **(E)** Proportions of GABAergic, glycinergic and mixed phenotypes of simulated mIPSCs as function of α. Each point in **(B–D)** represents the average value of the distribution for an α value and the color blend is a linear function of α between pure GABA (3 mM, blue, a = 0) and pure glycine (3 mM, orange, α = 1). Figure 4A has been reproduced from Aubrey et al. (2007).

In Figure 4 and Supplementary Figure S3, we examined the distribution of mIPSC phenotypes that were simulated with a broad variation in [Gly]_peak_ and [GABA]_peak_ and/or GABA_A_ and GlyR. Normal distributions of [Gly]_peak_, [GABA]_peak_ and/or GABA_A_ and GlyR were generated using the Random Variate and NormalDistribution functions of Mathematica with [Gly]_peak_ = *N*[3α, (3α CV)^2^] and [GABA]_peak_ = *N*[3(1−α), (3(1−α)CV)^2^]. Accordingly, the average [AAN]_peak_ was constant (3 mM) in all simulations.

### Ethics Statements

This study was carried out in accordance with the recommendations of guidelines of the Centre National de la Recherche Scientifique. The protocol was approved by the CNRS under number 02235.02 of the general agreement C750520.

## Results

### Identification of mIPSCs From Individual Terminals Using a Combination of Loose-Patch and Whole-Cell Electrophysiology in Cultured Spinal Neurons From GlyT2-eGFP Mice

We combined the spinal cords of wild-type and GlyT2-eGFP (5–10%) embryos (Zeilhofer et al., 2005) and made low-density cultures of eGFP-expressing neurons that were used for electrophysiological experiments at DIV14–21. When viewed under fluorescent light, we observed thin, parallel neurites with beaded varicosities (1.05 ± 0.2 μm, *n* = 30), suggestive of axons (Figure 1A). The GlyT2-eGFP^+^ beaded varicosities rapidly accumulated the styryl dye FM 4–64, a marker of cycling presynaptic vesicles (Figure 1B), confirming that they correspond to presynaptic terminals.

Next, we voltage-clamped (V_H_ = −70 mV) the cell body of an eGFP^−^ neuron that appeared to have direct contact with one or a few eGFP^+^ varicosities at or near to its soma, and recorded WC mIPSCs in the presence of TTX (Figure 1C). WC mIPSCs are heterogeneous because they correspond to the postsynaptic neurons response to the spontaneous release of single synaptic vesicle at one of its many inhibitory terminals (Hubbard et al., 1967). To identify the specific subset of mIPSCs that originated from a single GlyT2-eGFP^+^ varicosity, we simultaneously recorded extracellular mIPSCs from a second LP recording electrode (V_H_ = 0 mV) placed directly over a single synapse, identified by its eGFP^+^ varicosity, located at or near to the soma of the recorded neurons (Figure 1C; Forti et al., 1997). Single-synapse mIPSCs were identified from whole cell mIPSCs (inward) that were time- and amplitude-locked to extracellular LP mIPSCs (outward, Figures 1C,D). LP-mIPSCs were never detected if the LP-pipette was not centered precisely on the GlyT2-eGFP^+^ varicosity, consistent with the prediction that the LP-pipette will detect less than 1% of currents that originate outside the LP pipette (Forti et al., 1997) and suggestive that detection of current from two independent synases is unlikely.

### GABA- and Glycine-Only mIPSCs Events Were Defined Based on Their Peak Amplitude to Charge Relationship

Although GABA and glycine activate ligand-gated chloride channels with distinct biophysical and pharmacological properties, GABAergic and glycinergic mIPSCs are difficult to separate because of their overlapping receptor kinetics (Burzomato et al., 2004; Labrakakis et al., 2014). We used flunitrazepam, a positive allosteric modulator of GABA_A_ receptors, to selectively slow the decay of GABAergic mIPSCs and allow a crude separation of GABA vs. glycine mIPSCs based on their peak amplitude and charge transfer (Figures 2A,B). Nevertheless, the two mIPSC populations were highly variable both within and between neurons (Figure 2C; *n* = 15 cells, 600 mIPSC events each). The variability is attributable to the presence of multiple independent inhibitory connections with potential differences in: receptor numbers, receptor subunit composition, synaptic architecture, as well as synaptic vesicle content (Ropert et al., 1990; Frerking et al., 1995; Auger and Marty, 1997; Nusser et al., 1997) and dendritic filtering (Gardner et al., 1999). This variability was not reduced by analyzing data from neurons prepared from the same animals, or on the same number of days *in vitro* (data not shown). Linear regressions of the peak amplitude vs. charge data and 95% prediction intervals frame the GABA and glycine mIPSC populations and were used to classify pure and mixed phenotypes (Figure 2C). When we recorded whole cell mIPSCs in the absence of strychnine and SR 95531, and selected mIPSC events with clear biphasic decay currents characteristic of mixed GABA/glycine cotransmission (Jonas et al., 1998; Lu et al., 2008), these events mapped in-between the GABA and glycine mIPSC prediction intervals on graphs of peak amplitude vs. charge (Figure 2D) or within the GABA mIPSC prediction intervals (Figure 2E). Thus, the linear regression and 95% prediction intervals of the glycine peak to charge data provides functional limits to define glycinergic and mixed mIPSCs. In contrast, the GABAergic mIPSC data was not able to differentiating between mixed and GABAergic mIPSCs, especially when mixed mIPSC currents had a large GABA component (Figure 2E).

To explore all possible phenotype outcomes in response to variable GABA and glycine vesicular release, we simulate glycinergic, GABAergic and mixed mIPSCs with activation kinetic models for GABA_A_R and GlyR that have been previously established (Burzomato et al., 2004; Labrakakis et al., 2014; Figures 3A,B). We limited our simulations to the simplest, linear substitution of GABA by glycine, keeping a constant [neurotransmitter release] (AAN_peak_ = 3 mM, τ_r_= 0.7 ms (Beato, 2008), Figure 3C). Therefore, increasing α potentiates the glycine component and decreases the GABA component of mixed mIPSCs (Figures 3D–F) in such a way that the increase in peak amplitude may compensate for the reduction in charge transfer, thus preserving the strength of synaptic inhibition (Figure 3G).

To map a more realistic distribution of inhibitory phenotypes, we plot the peak-charge amplitude distribution of simulated mIPSCs for a randomly generated number of postsynaptic receptors and peak concentration transients (see “Materials and Methods” section). First, we plot the smooth density histograms for 200 simulated mIPSCs when there is a unique, pre- or post-synaptic source of variability with a coefficient of variation of 0.2 (CV_R_ = 0.2 or CV_AAN_ = 0.2, Supplementary Figure S3). Supplementary Figure S3 illustrates that a lower variability of the GABA mIPSCs is expected when α values are low, because GABA_A_R receptors are almost saturated by 3 mM GABA transient (EC_50_ = 0.81 mM for GABA_A_R and 2.68 mM for GlyR, with comparable apparent hill-coefficients of 2.17 and 2.06, respectively). Then we map the distribution of 500 randomly simulated mIPSCs with average α values ranging from 0 (pure GABA) to 1 (pure Glycine) by 0.05 step increments (Figure 4A), with high pre- (CV_R_ = 0.4, Figure 4B) and post- (CV_AAN_ = 0.4, Figure 4C) synaptic variability. By definition, the simulated mIPSCs are mixed and have two components when 0 < α < 1 and would therefore be arbitrarily qualified as mixed, even if one component is small and the mIPSC resembles pure GABAergic or pure glycinergic events. Therefore, we delimit three areas in the charge-peak amplitude plot (Figure 4A) for the attribution of GABAergic (blue area), glycinergic (orange area) and mixed phenotypes (green area), based on the 95% prediction from the linear regression of the experimental values for charge-peak amplitude. The average peak-charge amplitudes for each α values follow the model predictions (Figure 4D) and the repartition of mIPSC phenotypes as function of α (Figure 4E) shows that 10% GABA release (α = 0.9) reduces the glycinergic component of the mixed mIPSCs by 50%, whereas in contrast a similar reduction in GABAergic phenotypes is not achieved until α = 0.3. This finding is consistent with our experimental data (Figures 2D,E) and indicates that mixed mIPSCs with small glycine components are likely to be characterized as a GABAergic phenotype. Overall, the model confirms that neurotransmitter release by vesicles containing higher proportions of glycine than GABA will evoke mixed mIPSCs that have intermediate peak amplitude-charge relationships, falling in-between the GABA and glycine prediction intervals defined by our experimental data. When α = 0.45–0.85 the model predicts that mixed phenotypes will be the dominant phenotype, with <20% of mixed events being mischaracterized.

### A Combination of Mixed and Glycinergic mIPSCs Were Detected at Some Individual Terminals

**Figure 5:**
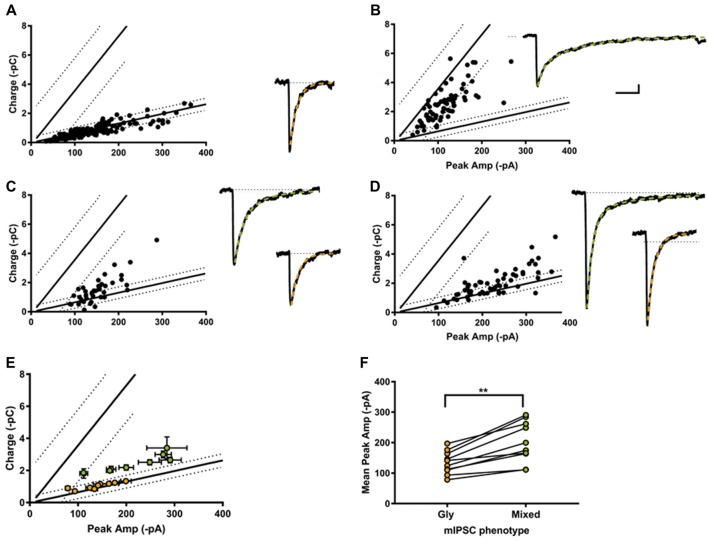
Most individual inhibitory synapses had a combination phenotype comprised of both glycine and mixed mIPSCs. Examples of single synapse peak amplitude-charge relationships (left) and average mIPSCs (10 events each, right) with their decay current fitted with a single (orange) or double exponential (green). 4/18 of the recorded synapses were purely glycinergic **(A)**, and 5/18 were purely mixed **(B)**. The other 9/18 synapses displayed a combination of mixed and glycine mIPSCs **(C,D)**. **(E)** Average peak current-charge relationship of the glycine (orange) and mixed (green) mIPSCs identified in the nine combination synapses. **(F)** Glycine mIPSC peak amplitudes were always smaller than the mixed mIPSC peak amplitude, as expected from a compound GABA+glycine mIPSC event.

Individual-terminal mIPSCs were recorded in the absence of strychnine and SR 95331, and analyzed when the peak amplitude and charge values were measurable in ≥25 LP-linked mIPSCs. We classified the mIPSC phenotype from 18 individual terminals. In 4 of the 18 synapses only glycine mIPSC were detected (Figure 5A), with a reduced co-efficients of variation (CV) compared to glycine mIPSCs from whole cell recording (0.42 ± 0.5, range 0.30–0.51; and 0.65 ± 0.05, range 0.25–0.86, respectively). The rest (14/18) were cotransmission terminals, with a substantial proportion of mixed mIPSCs. According to our classification system, we found that 36% (5/14) of the cotransmission terminals we sampled had a homogenous mixed mIPSC phenotype as hypothesized (Figure 5B). The majority (64%, 9/14) had a heterogeneous phenotype made up of a combination of mixed and glycine-only mIPSCs (Figures 5C,D). In these combination synapses, the mixed eIPSCs were always larger than glycine-only mIPSCs, as predicted for an additive current, and the mixed current was carried primarily by glycine (Figures 5E,F). Examination of the raw mIPSCs currents grouped into their designated phenotypes (averaged from 10 consecutive events), demonstrates that phenotypically glycinergic mIPSCs have characteristic fast monophasic decay kinetic (Figures 5A,C,D). In contrast, mIPSCs events that fell into the mixed or GABA phenotype regions all had a discernable biphasic kinetics (Figures 5B–D) indicating this population corresponded to mixed events. Together, these data supports the identification of distinct mixed and pure mIPSC phenotypes in combination synapses.

## Discussion

### Heterogeneity of GABA/Glycine Vesicular Content as a Presynaptic Source of mIPSC Variability

Because identifying the glycine and GABA components in individual mixed IPSCs is not straightforward, we previously used a giant-synapse, made from a secretory BON cell and a sniffer HEK cell, to sample all vesicular phenotypes when a VIAAT-expressing BON cell had access to both neurotransmitters (Aubrey et al., 2007). In this cellular model, sniffer HEK cells expressed GABA and glycine receptors that had cationic and anionic permeability respectively, thus GABA- and glycine-evoked currents had opposite polarities and could be identified unambiguously (Aubrey et al., 2007, Supplementary Figures S1A–C). Surprisingly, the distribution of pure and mixed events from individual co-releasing BON cells suggested heterologous vesicular GABA/glycine content (Aubrey et al., 2007, Supplementary Figure S1C), as reported in acutely dissociated spinal cord neurons (Katsurabayashi et al., 2004). We can excluded a significant contribution of the sniffer cell in the BON/HEK model because scanning across the entire sniffer HEK cell surface with brief iontophoretic applications of a fixed [GABA] and [glycine] evoked stable biphasic currents (Supplementary Figures S1D,E), again supporting heterogeneous vesicular content that favors glycine- or GABA-like mIPSCs.

In the present work, we combined LP extracellular recordings with WC recordings to sample mIPSCs from single inhibitory varicosity in spinal cord cultures from GlyT2:EGFP mice. We have shown previously that evoked IPSCs from GlyT2-expressing neurons are predominately mixed, with a larger glycinergic component (Rousseau et al., 2008). As expected, we detected mixed mIPSCs in the majority (78%) of GlyT2-GFP^+^ synapses sampled and found that most mixed GABA/glycine synapses signaled with a combination of pure and mixed mIPSCs. This phenotypic heterogeneity may have a presynaptic origin that results from variability in the accumulation and packaging of GABA and glycine in VIAAT-containing synaptic vesicles, similar to the BON/HEK model system (Aubrey et al., 2007; Supplementary Figure S1), however we cannot rule out a postsynaptic origin because inhibitory synapses are dynamic structures (Choquet and Triller, 2013), with glycine and GABA_A_ receptors being constantly trapped or exchanged between gephyrin containing nano-domains (Calamai et al., 2009; Maric et al., 2014; Tyagarajan and Fritschy, 2014; Alvarez, 2017; Pennacchietti et al., 2017). In addition, varicosities are larger structures than the small en passant boutons of typical central synapses that may contain multiple active zones facing postsynaptic clusters with different receptor compositions (Nusser et al., 1997; Biró et al., 2006; Lévi et al., 2008). When we generated simulated mIPSCs including a pre- and a post-synaptic source of variability (Supplementary Figure S3), we found that both were able to recapitulate the mostly glycinergic mIPSC distributions observed experimentally. We did not attempt to differentiate the source of variability any further in the present work.

### mIPSC Detection Bias

The distribution of mIPSC phenotypes, which is based on their peak to charge relationship, indicates that single varicosities predominantly signal with glycine in these cultures (α ≥ 0.7). This dominant glycinergic phenotype was anticipated because: 1. all the synapses sampled had GlyT2-eGFP^+^ presynaptic varicosities. 2. Glycine mIPSCs have faster kinetics and larger peak amplitude than GABA mIPSCs. As a result, 3. time-integration of mIPSCs was more consistently resolved for glycine events than for GABA events because often GABA mIPSCs that did not return to baseline before a subsequent mIPSC occurred and thus could not be reliably integrated. 4. Mixed mIPSCs with a high or low α-values are likely to be mis-classified, although mixed mIPSCs with small GABA components (α > 0.9) were more easily detected than those with small glycine components (α < 0.3). 5. Finally, mIPSCs with larger peak amplitudes, typically carried by glycine currents in these cultures, were more likely to be detected by the low resistance, LP recordings.

### A Model to Study Cotransmission

We modeled mixed mIPSCs as the summation of the GABA_A_Rs and GlyRs mediated currents activated by a fast synaptic transient of GABA and glycine. Each randomly generated transient aims to represent the possible content of one VIAAT expressing vesicle. As the time course of glycine and GABA in the synaptic cleft have been thoroughly modeled (Overstreet and Westbrook, 2003; Beato, 2008), we used a fixed average peak concentration of 3 mM and a synaptic transient time-constant of 0.7 ms for the simulation. We fixed the peak-concentration transient because the amount of neurotransmitter stored in a vesicle may be limited by VIAAT thermodynamics, VIAAT kinetics or set by other vesicular factors (Edwards, 2007).

We used kinetic models and rate constants well-established for both receptors (Burzomato et al., 2004; Labrakakis et al., 2014), and assumed for simplicity that glycine and GABA act independently, although it has been shown that GABA/glycine corelease accelerates the kinetics of glycinergic IPSCs in pure glycinergic synapses (Lu et al., 2008). The distribution of randomly generated simulated mIPSCs on the charge-peak amplitude plot provided a graphical overview of all transitions between pure GABAergic and pure glycinergic phenotypes when the proportion of glycine in the neurotransmitter transient is sequentially increased. We operationally distinguished pure and mixed phenotypes for individual mIPSCs based on their location on three experimentally determined areas in the charge-peak amplitude plot. The distribution of “pure” GABAergic, mixed and “pure” glycinergic phenotypes shows that “pure” glycinergic phenotype is only compatible with pure glycine release since α = 0.95 (about 150 μM GABA and 2.85 mM Glycine) is enough to evoked mixed phenotypes. In contrast “pure” GABAergic phenotypes are reported up to α = 0.2 (about 2.4 mM GABA and 0.6 mM glycine), indicating that minor glycine corelease may more easily go unnoticed than minor GABA corelease. This asymmetry reflects the apparent saturation of GABA_A_Rs with a 3 mM GABA transient.

### Functional Roles for GABA/Glycine Corelease

GABA/glycine co-signaling is habitually thought to be a property of immature inhibitory synapses in the spinal cord, brainstem and granular layer of the cerebellum that diminishes during postnatal development, with few exceptions (Inquimbert et al., 2007; Bhumbra et al., 2012). Indeed mixed GABA/glycine cotransmission was initially detected in juvenile rat spinal and brainstem motoneurons (Jonas et al., 1998; O’Brien and Berger, 1999; Russier et al., 2002) and lamina I and II of the spinal cord dorsal horn (Keller et al., 2001). Furthermore, a critical shift toward pure glycinergic transmission takes place during the postnatal maturation in spinal cord locomotor centers (Gao et al., 2001) and brainstem auditory nuclei where the large amplitude and rapid decay of glycinergic IPSCs provide the microsecond precision needed for the processing and the coding of acoustic information (Brand et al., 2002; Nabekura et al., 2004).

However, corelease with or without cotransmission persists in adults at some synapses, suggesting a continuing specific need for each neurotransmitter. In the superficial lamina of the spinal cord, GABA and glycine cotransmission is replaced by pure glycinergic transmission after P23 (Keller et al., 2001). Vesicular corelease persists in into adulthood however, and coreleased GABA influences the release probability by acting at presynaptic GABA_B_R (Chéry and de Koninck, 2000). Furthermore, extrasynaptic GABA_A_ receptor signals can be detected at higher stimulation intensities (Chéry and de Koninck, 1999). At physiological temperatures evidence suggests that GABA and glycine corelease may not contribute to spillover and tonic inhibition in the dorsal horn of the spinal cord, as the diffusion of GABA and glycine out of the synaptic cleft is readily controlled by transporters in this region (Mitchell et al., 2007). Nevertheless, spillover of synaptically released glycine has been shown to potentiate the NMDA responses in the superficial dorsal horn of the spinal cord, by increasing the occupancy of the NMDA receptor coagonist site (Ahmadi et al., 2003). The same developmental pattern of inhibitory transmitter use has been reported in the rat MNTB, where GABA/glycine cotransmission is detected until P9–P12 after which time pure glycinergic transmission dominates (Awatramani et al., 2005). Again vesicular corelease persists in older animals, and coreleased GABA has been shown to shorten glycinergic mIPSC decay times here (Lu et al., 2008). In the central nucleus of the inferior colliculus of the auditory midbrain, GABA and glycine cotransmission persists in brain slices from P19 to P35 mice. Coreleased GABA and glycine evoke IPSCs with nearly identical amplitude and time course, suggesting that GABA and glycine are operationally fully interchangeable in this nucleus, thus securing inhibitory signaling by redundancy (Moore and Trussell, 2017). Finally, in the cerebellum of juvenile rats, inhibitory corelease occurs between Golgi cells and vestibulocerebellar Unipolar Brush Cells (Dugué et al., 2005; Rousseau et al., 2012), and once again evidence suggests that the transmission phenotype is under postsynaptic control. Interestingly, the phenotype of transmission in this region is not fixed developmentally, but seems to be coordinated with the phenotype of the glutamatergic input onto the Unipolar Brush Cell (Rousseau et al., 2012).

Our data suggests that a dynamic vesicular GABA/glycine balance at individual synapses may contribute to the tuning of phasic synaptic inhibition in spinal neurons. If the origin of the variability is indeed the neurotransmitter content in presynaptic vesicles, then glycine IPSC kinetics (Lu et al., 2008) and the probability of transmitter corelease (where GABA_B_ receptors are expressed (Chéry and de Koninck, 1999, 2000) would also contribute to this fine tuning.

### Possible Mechanisms for Vesicular GABA/Glycine Variations at Single Terminals

The mechanisms that specify or regulate the vesicular GABA/glycine content at mixed synapses are not well understood. As previously discussed, changes to GABA (Mathews and Diamond, 2003; Wang et al., 2013) and glycine supply/resupply (Rousseau et al., 2008; Apostolides and Trussell, 2013; Ishibashi et al., 2013) can strongly shift the GABA/glycine balance and alterations in IPSC characteristics can be observed within minutes. Indeed, metabolic alteration such as these have been implicated in disease states including chronic pain (Coull et al., 2003; Imlach et al., 2016; Takazawa et al., 2017) and amyotrophic lateral sclerosis (Medelin et al., 2016).

With an apparent lower affinity for glycine than for GABA (about 25 and 6 mM, respectively), VIAAT uptake is likely to be rate limiting and a major source of variability for vesicular loading (Burger et al., 1991; Gasnier, 2000; Edwards, 2007; Farsi et al., 2016; Takamori, 2016). In addition, VIAAT has presumably the lowest driving force for uphill transport among the vesicular transporters (Edwards, 2007), being coupled to the exchange of a single H^+^ (Hell et al., 1991; McIntire et al., 1997; Farsi et al., 2016) and with smaller protonmotive force (Egashira et al., 2016). If VIAAT kinetic is limiting for filling vesicles, as suggested by a slow recovery from synaptic depression (Katsurabayashi et al., 2004; Rousseau et al., 2008; Wang et al., 2013; Yamashita et al., 2018), then the initial vesicular content may continue to change as VIAAT exchanges a fast filling neurotransmitter (presumably GABA) with cytosolic glycine, providing opportunity for alterations in the transient neurotransmitter composition.

Other potential mechanisms include protein-protein interactions between the transmitter supply machinery and different populations of vesicles (Jin et al., 2003), differential modulation of the pool of cycling vesicle in response to presynaptic GABA or glycine supply (Mathews and Diamond, 2003; Wang et al., 2013; Truckenbrodt et al., 2018) or differential modes of GABA and glycine uptake by VIAAT (Aubrey et al., 2007). Given the diverse and highly regulated roles of the presynaptic terminal, this list is in not exhaustive.

In summary, this work adds to a growing body of evidence that suggest that the presynaptic terminal and neurotransmitter corelease can significantly regulate synaptic transmission. We present evidence that the GABA/glycine balance at individual co-releasing terminals is variable, and predict these variations would serve to fine tune the timing of inhibition and the integration of sensory inputs. Given the complexity and high level of control the presynapse has over synaptic vesicle release (Rizzoli and Betz, 2004; Edwards, 2007; Südhof, 2012), small, dynamic alternations in the balance of GABA/glycine signaling are possible and likely to contribute to synaptic plasticity.

Cotransmission is known to shape synaptic plasticity of other brain regions (Vaaga et al., 2014; Tritsch et al., 2016). For example, in the lateral habenula the GABA component of GABA/glutamate co-releasing inputs from the basal ganglia are selectively reduced in an animal model of depression. This deficiency is restored following sustained treatment with the antidepressant citalopram (Shabel et al., 2014). A similar reduction in the GABA component of mixed GABA/glutamate release has been observed in the same region in animal models of cocaine withdrawal, where it is attributed to a selective decrease in VIAAT expression in vesicles (Meye et al., 2016).

## Author Contributions

SS and KA conceived and planned the experiments. KA carried out the experiments and their analysis. SS carried out the modeling and simulations. SS and KA contributed to the interpretation of the results and wrote the manuscript.

## Conflict of Interest Statement

The authors declare that the research was conducted in the absence of any commercial or financial relationships that could be construed as a potential conflict of interest.
