# Supplementary material for: Heterogeneous Signaling at GABA and Glycine Co-releasing Terminals
Source: Front Synaptic Neurosci. 2018 Nov 6;10:40. doi: 10.3389/fnsyn.2018.00040 (PMC6232519; doi:10.3389/fnsyn.2018.00040)
Supplement: Supplementary file 1 [file Presentation_1.pdf]

*Supplementary Material*

# Heterogeneous signaling at GABA and glycine co-releasing terminals

**Karin R. Aubrey[1,2,3*] & Stéphane Supplisson[1]**

*** Correspondence:** karin.aubrey@sydney.edu.au or stephane.supplisson@ens.fr

## Supplementary Figures

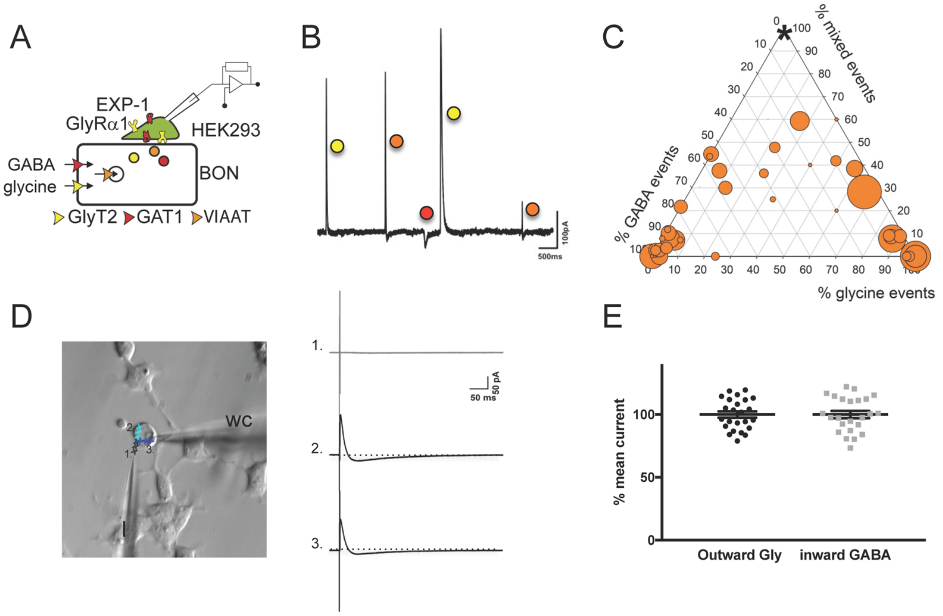


**Supplementary Figure S1. Heterogeneous glycine and GABA release from a giant sniffer cell synapse.**

(**A**) A model of giant-inhibitory-synapse was constructed by manually pressing a HEK 'postsynaptic' cell transfected with glycine and GABA receptors (GlyR and EXP-1) against a secretory BON 'presynaptic' cell transfected with VIAAT and membrane transporters (GAT-1 and GlyT2) and used to detect single vesicle release from a co-releasing pre-synaptic terminal (from Aubrey et al., 2007). (**B**) The current trace shows a combination of a purely GABAergic inward current (red circle), purely glycinergic outward currents (yellow circles) as well mixed biphasic currents (orange circles)

from a single BON/HEK giant synapse. (C) proportion of all event phenotypes (GABAergic, glycinergic and mixed) recorded from individual BON cells (from Aubrey et al., 2007). The circle diameters are proportional to the square root of the number of events and the * symbol indicates the expected proportion of mixed events when BON cells have access to both GABA and glycine (**D**). To test if the HEK sniffer cell expressed clusters of glycine and GABA receptors evenly across the cell membrane, we iontophoretically applied rapid synaptic-release-like puffs of a fixed concentration of GABA and glycine across the HEK membrane. (**E**). The GABA/glycine amplitude and ratio remained constant across the surface of HEK:GlyR:EXP-1 ‘postsynaptic’ cells, indicating that the variability observed in B-C. can not be attributed to receptor clusters and most likely reflects distinct inhibitory neurotransmitter content in the released vesicles.

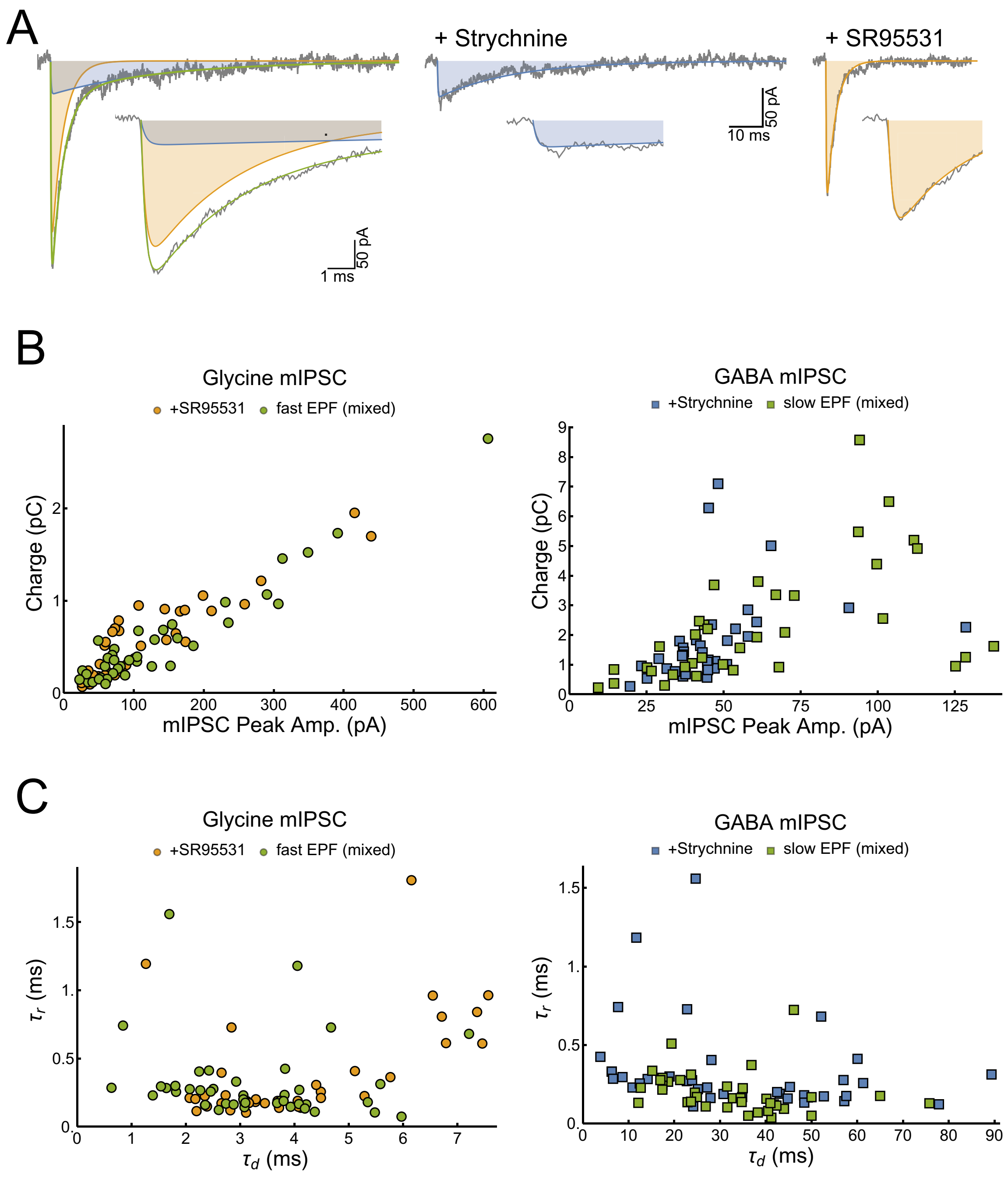


**Supplementary Figure S2. Delineation of the glycinergic and GABAergic components of mixed IPSCs. Comparison with pharmacologically isolated pure Glycinergic and GABAergic mIPSCs.**

**(A)** Representative traces of mixed and pure GABAergic and glycinergic mIPSCs recorded in control condition or in the presence of strychnine and SR95531, respectively. Pure mIPSCs were well described with a piecewise-defined single exponential product function (EPF, Jonas et al 1993, Rahman et al 2013):

$$I_t = \begin{cases} 0 \cdots\cdots\cdots\cdots\cdots\cdots\cdots\cdots\cdots\cdots t < t_0 \\ A\left(1 - e^{\frac{-(t-t_0)}{\tau_r}}\right) e^{\frac{-(t-t_0)}{\tau_d}} \cdots\cdots\cdots t \geq t_0 \end{cases}$$

where A is the amplitude, $\tau_r$ is the rising time constant, $\tau_d$ is the decay time constant and $t_0$ the mIPSC onset time. Fits were done on background subtracted current using Excel Solver.

Mixed mIPSCs were fitted with the sum of a fast and slow EPFs with a common rise time, thus limiting the number of free parameters:

$$I_t = \begin{cases} 0 \cdots\cdots\cdots\cdots\cdots\cdots\cdots\cdots\cdots\cdots\cdots\cdots\cdots\cdots\cdots\cdots\cdots\cdots t < t_0 \\ A_{Gly}\left(1 - e^{\frac{-(t-t_0)}{\tau_r}}\right) e^{\frac{-(t-t_0)}{\tau_{d,fast}}} + A_{GABA}\left(1 - e^{\frac{-(t-t_0)}{\tau_r}}\right) e^{\frac{-(t-t_0)}{\tau_{d,slow}}} \cdots t \geq t_0 \end{cases}$$

The mIPSC peak amplitudes ($I_{peak}$) and charges (Q) of each component were calculated according to the equations:

$$I_{peak} = A \left(\frac{\tau_r}{\tau_d + \tau_r}\right)^{\tau_r/\tau_d} \left(1 - \frac{\tau_r}{\tau_d + \tau_r}\right)$$

$$Q = A\ \tau_d \left(1 - \frac{\tau_r}{\tau_d + \tau_r}\right)$$

**(B)** Peak amplitude and charge of pure mIPSCs (left panel: GABA, blue circles, n = 36; right panel: glycine, orange circles, n = 38) and mixed mIPSCs with the slow (left panel: green open circles, n = 36) and fast (right panel: green open circles, n = 38) EPFs. We hypothesized that both methods should give a similar distribution of the peak amplitudes and charges as all mIPSCs were recorded from the same postsynaptic cells, and run the non-parametric Conover square-ranks test using Mathematica standard statistical functions. However, the Conover test rejected the null hypothesis for equal distribution of GABA peak amplitudes ($p = 0.00011$) and charges ($p = 0.02$). In contrast, the null hypothesis was not rejected for glycine peak amplitudes ($p = 0.44$) and charges ($p = 0.12$), indicating a similar dispersion for both methods.

**(C)** the relationship between the rise time constant and the decay time constant for glycine and GABA shows little evidence for significant dendritic filtering.

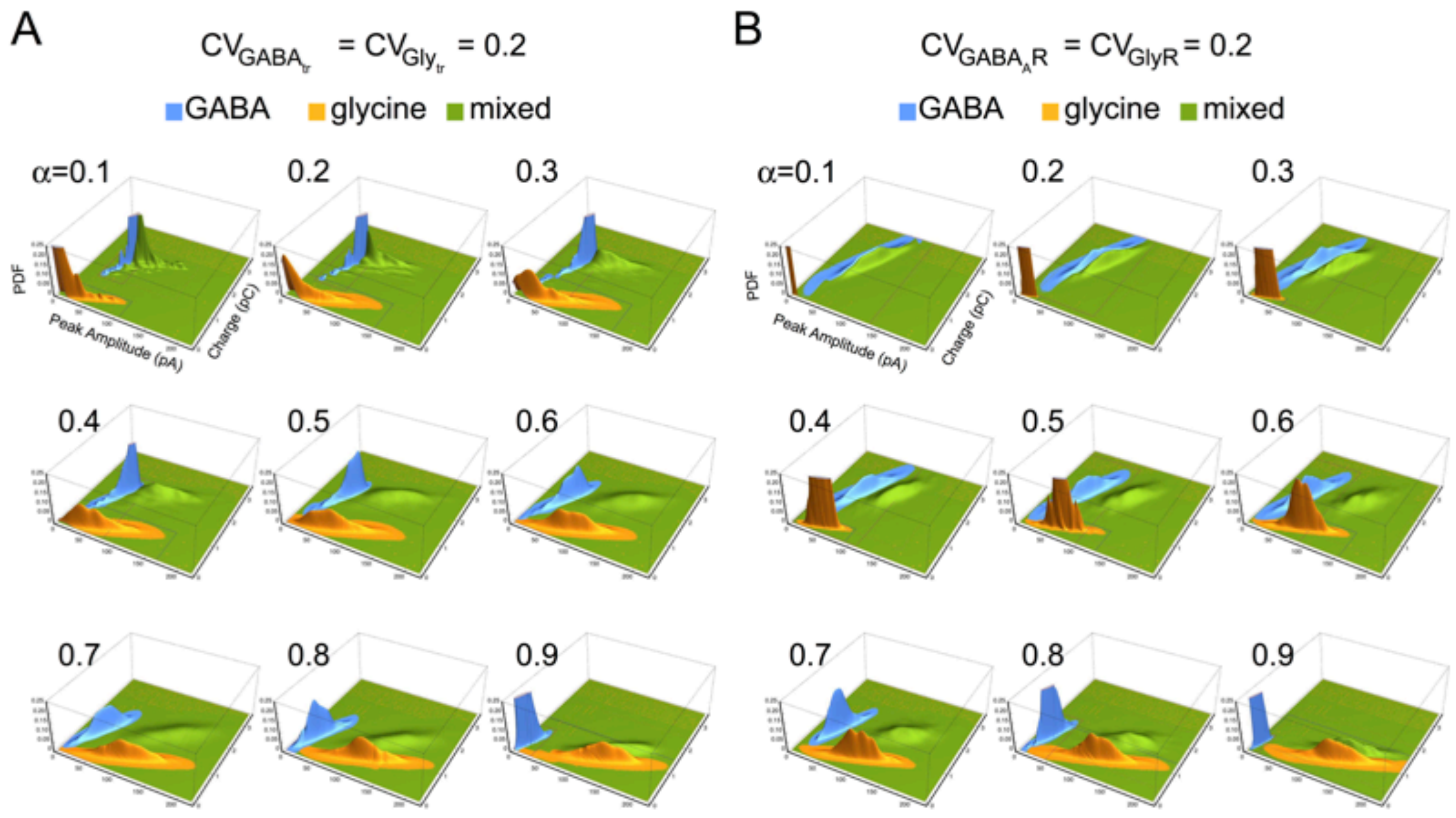


**Supplementary Figure S3. Effect of pre- and post- synaptic source of variability on the charge-amplitude distribution of mixed mIPSCs.** (**A-B**) Smooth density histograms of the charge-peak amplitude for numerical simulations of mixed mIPSCs with a presynaptic (**A**) or a postsynaptic (**B**) source of variability (200 simulations for each panel). The frequency in the z axis is clipped at 0.25. The $\alpha$ value increased from 0.1 to 0.9 by increment of 0.1 as indicated on each panel. (**A**) The transient concentration was randomly generated from normal distributions with $CV_{AAN} = 0.2$. (**B**) The numbers of post synaptic receptors were randomly generated from normal distributions with $CV_R = 0.2$.
